# Comparison of two-stage open reduction and internal fixation and single-stage external fixation for complex pilon fractures: a randomized controlled trial

**DOI:** 10.1007/s00264-025-06682-2

**Published:** 2025-11-13

**Authors:** Mohamed Osama Eissa, Mootaz Fouad Thakeb, Salah AbouSeif, Tamer A. Fayyad, M. A. Alkersh, Mohamed A. ElGebeily, Ahmad Saeed Aly, Mostafa M. Baraka

**Affiliations:** Department of orthopedic surgery, Ain Shams University, Cairo, Egypt

**Keywords:** Pilon fracture, External fixation, Staged ORIF, Damage control

## Abstract

**Purpose:**

To compare functional and radiological outcomes between two stage ORIF and single stage external fixation for complex pilon fractures.

**Methods:**

Prospective, single-center randomized controlled trial at a Level I trauma facility (April 2021–April 2023). Sixty skeletally mature patients with AO/OTA 43-C pilon fractures unsuitable for primary ORIF were randomized to two-stage ORIF (control group) or single-stage limited internal fixation with external fixation (LIFEF) (treatment group). Minimum follow-up was 24 months. The primary outcome was the AOFAS score at final follow-up. Secondary outcomes included time to union, time to return to work, ankle range of motion (ROM), fracture-related infection (FRI), bone-healing complications (nonunion, malunion, delayed union), post-traumatic osteoarthritis (PTOA), and need for secondary procedures.

**Results:**

All 60 patients completed follow-up. Compared with LIFEF, two-stage ORIF achieved higher AOFAS scores (85 ± 9 vs. 77 ± 10; *P* = 0.006), earlier return to work (7 ± 1.5 vs. 10 ± 3 months; *P* < 0.001), and shorter time to union (17 ± 3.6 vs. 19 ± 3.5 weeks; *P* = 0.02). Groups did not differ in quality of reduction (*P* = 0.14), ankle ROM (*P* = 0.10 and 0.058 for dorsiflexion and plantarflexion), FRI (*P* = 0.69), PTOA (*P* = 0.64), or bone-healing complications (nonunion, delayed union, malunion; *P* = 0.24, 0.39, 0.39).

**Conclusion:**

Two-stage ORIF provided superior functional outcomes and faster recovery (earlier union and return to work) compared with LIFEF, with similar reduction quality and complication rates. These findings support two-stage ORIF as the preferred strategy for AO/OTA 43-C pilon fractures with soft-tissue compromise.

**Registry:**

ClinicalTrials.gov, NCT05141227, Registration date: 29 July 2021.

## Introduction

Pilon injury accounts for about five to ten% of all tibial fractures, and for less than 1% of lower extremity injuries [1]. Among all pilon fractures, about 30% are complex pilon fractures (AO/ OTA 43-C type) caused by high-energy injuries. Most complex pilon fractures are associated with severe soft tissue injuries, making the treatment challenging [2]. Several methods have been advocated to manage complex pilon fractures, but an optimal fixation technique remains controversial. With the accumulation of surgical experience and the development of surgical techniques, two-stage ORIF and limited internal fixation combined with external fixation (LIFEF) were established, and these two methods are now widely advocated for the treatment of comminuted tibial pilon fractures [3]. There is currently no consensus regarding the superiority of either method in treating complex pilon fractures especially AO/ OTA 43-C type in terms of clinical, radiological, and functional outcomes. We hypothesis that limited internal fixation with external fixation is still valid option in patients with significant soft tissue injuries.

## Materials and methods

A prospective randomized controlled comparative clinical trial conducted at tertiary trauma centre was designed to investigate the optimal treatment approach for complex pilon fractures with significant soft tissue injury. Following approval from the ethics and research committee (FWA A00017585 Approval FMASU MD 205/2021), written informed consent was obtained from all participating patients. From April 2021 to April 2023, a total of 60 patients with these fractures were enrolled, with 30 patients assigned to each of two study groups.

Control group consisted of patients treated with a two-stage ORIF, while treatment group comprised patients treated with LIFEF. Patients were followed up for minimum 24 months postoperatively. Patients were randomly allocated to one of two groups using a computer-generated randomization sequence created by an independent statistician. Patients were randomly assigned in a one to one ratio to one of the two groups. Allocation concealment was maintained by using sequentially numbered, opaque, sealed envelopes. The envelopes were opened only after the patient had provided informed consent and immediately before the surgical intervention.

Only skeletally mature patients with AO/OTA 43-C type pilon fractures, either closed or with open Gustilo grade I /II fractures, and with compromised soft tissue were included. Patients with ipsilateral lower limb fractures, presenting more than 24 h after injury, failed ankle fixation, pathological fractures, or pre-existing symptomatic ankle arthritis were excluded from the study.

Both patients and surgeons involved in the study were aware of the treatment allocations. While data analysts remained blinded to group allocation throughout the study, blinding of clinical outcome assessors was not feasible due to the visible differences between interventions (incisions/implants vs. external fixators). All patients included in the study were followed up and actively participated in the final analysis of study outcomes.

### Preoperative evaluation

All patients underwent a clinical assessment (trauma mechanism, smoking status, soft tissue and neurovascular status) and radiographic imaging (plain radiographs and CT with 3D reconstruction).

### Operative intervention

#### Control group

**Fig. 1:**
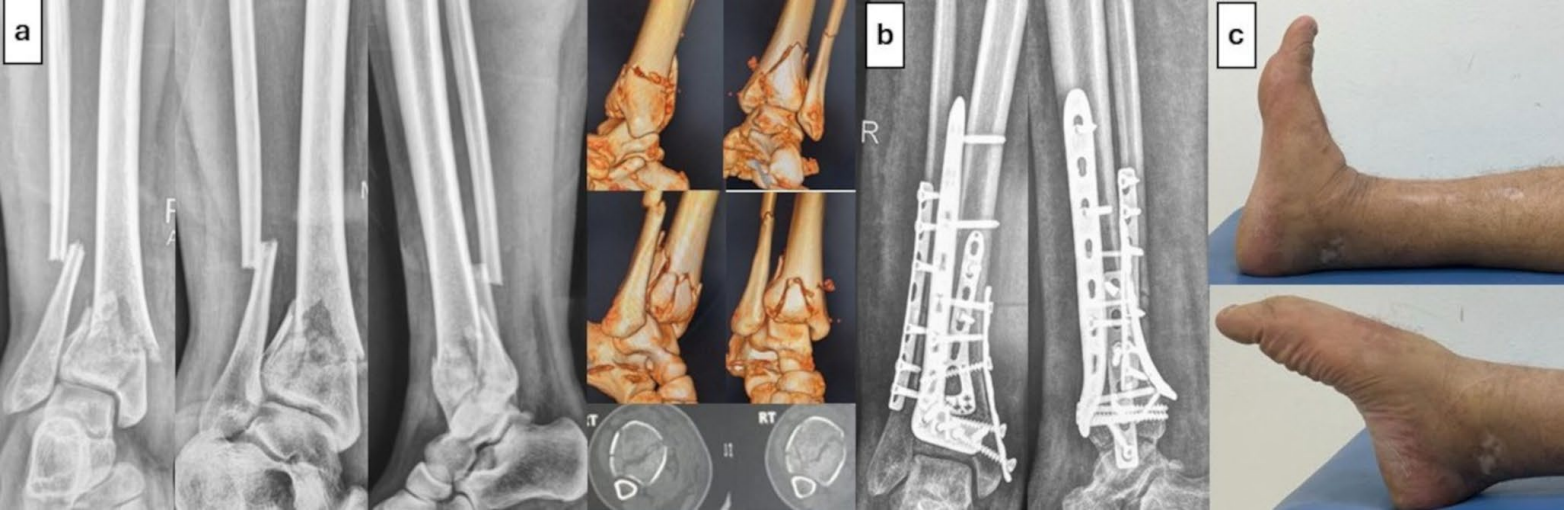
C3 Pilon fracture in 40 years old male, smoker, sustained as a result of RTA. (**a**) Preoperative images (**b**) 2 years after surgery (**c**) Ankle ROM

Temporary external fixation was applied early to restore alignment. Definitive fixation was performed after soft tissue recovery (skin wrinkling and re-epithelialization of fracture blisters). The choice of surgical approach and implants for definitive fixation primarily depended on the fracture pattern but was modified based on the condition of the soft tissue envelope. (Fig. 1)

#### Treatment group

**Fig. 2:**
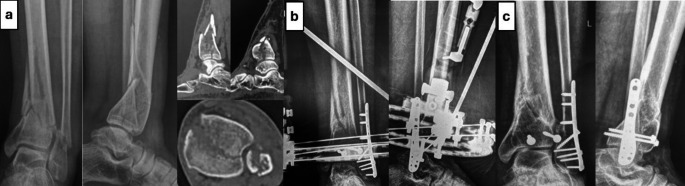
C3 Pilon fracture, 60 years old male, sustained as a result of falling from height. (**a**) Preoperative images (**b**) 1 month after surgery (**c**) 2 years after surgery

Fibular fixation was performed when indicated using either open or closed reduction methods and stabilized with a plate or intramedullary fixation, followed by articular reduction through mini-open or percutaneous techniques. Cannulated screws or olive wires were used as needed, and a ring fixator (ankle-spanning or sparing depending on stability of the distal fragment) was applied for definitive fixation. Wound closure was performed before the ring fixator was applied to minimize soft tissue tension and interference from the frame. (Fig. 2)

### Postoperative care and follow-up

Both groups received similar postoperative protocols including antiedema measures, non-weight bearing initially, and progressive mobilization according to fixation stability. Follow-up was conducted at regular intervals up to 24 months to assess functional, radiological, and complication outcomes.

### Outcome measures and assessment procedures

Primary and secondary outcomes were measured at baseline and each follow-up visit by two independent orthopaedic trauma surgeons. All radiographic assessments were performed by another two orthopaedic surgeons.

#### Primary outcome

- **American Orthopaedic Foot and Ankle Society (AOFAS) Score**: Assessed at the 24-month follow-up.

#### Secondary outcomes

- **Time to Return to Work**: Measured in months from date of definitive surgery to the date the patient resumed pre-injury occupational activities, verified through patient report and, when possible, employer confirmation.
- **Ankle Range of Motion (ROM)**: Measured at final follow-up using a standard goniometer with the patient seated and the knee flexed to 90°. Dorsiflexion and plantarflexion angles were recorded to the nearest degree. Measurements were taken three times and the mean value used.
- **Quality of Reduction**: Evaluated on postoperative and final follow-up radiographs using Burwell and Charnley criteria [4]. Measurements were performed on standardized weight-bearing anteroposterior, lateral, and mortise ankle radiographs.
- **Union Time**: Defined as the time from definitive fixation to radiographic evidence of cortical bridging in at least three of four cortices on orthogonal views, confirmed by both radiologists.
- **Post-Traumatic Osteoarthritis (PTOA)**: Graded on final follow-up non weight bearing radiographs according to the Takakura classification.
- **Bone Healing Complications:**
  *Nonunion*: No progression of healing by six months post-surgery.
  *Delayed union*: No progression of healing by three months post-surgery.
  *Malunion*: >five degree coronal angulation, > ten degree sagittal angulation, or > two mm intra-articular step-off [5].
- **Fracture-Related Infection (FRI)**: Diagnosed according to the 2018 international consensus definition (presence of sinus tract, purulent drainage, or confirmatory microbiology) [6].
- **Secondary Procedures**: Any unplanned reoperation related to the fracture or fixation, including hardware removal for infection, revision fixation, or bone grafting.

#### Data management and statistical analysis

The sample size was calculated based on the ability to detect a clinically significant difference in the primary functional outcome (AOFAS score) between the two groups. Based on prior studies, a mean difference of 8 points in AOFAS score was considered clinically significant, with an estimated standard deviation of ten points. Using G-power program, setting alpha error at 5%, power at 80% and effect size 0.80, a minimum of 26 patients per group was required. To account for possible dropouts and losses to follow-up, the sample size was increased to 30 patients per group, resulting in a total of 60 patients.

## Results

In this study, a total of 60 patients were included, with 30 patients in each group enrolled from April 2021 to April 2023. All patients were followed up for a minimum of 24 months. There were no losses or exclusions after randomization in either group. All randomized patients were included in the final analysis. The mean follow-up duration was 25.6 (range: 24–30) months in control group and 25.5 (range: 24–30) months in treatment group. There was no statistically significant difference observed between the two groups concerning demographic data, including age, gender, smoking status, the anatomical side affected, mode of trauma, fracture type (closed/open), fracture classification, or the presence of associated injuries (Table 1). Twenty-seven cases in treatment group (90%) were treated with an ankle-spanning Ilizarov frame, while three cases (10%) underwent an ankle-sparing Ilizarov frame. Additionally, 13 cases (43.3%) underwent limited internal fixation through mini-open approaches, and 17 cases (56.7%) had closed reduction of the articular surface followed by percutaneous fixation. Regarding fibular fixation, in control group, 20 cases were fixed with a plate, one case with intramedullary (IM) fixation, and nine cases had an intact fibula. In treatment group, four cases were fixed with a plate, 17 cases with IM fixation, three cases did not undergo fibular fixation, and six cases had an intact fibula. The mean time for conversion in control group was 11 days (range: 7–16 days) and the mean external fixation time in treatment group was 21 weeks (range: 16–34 weeks). (Table 2)

**Table 1:** Difference in demographic data in group A and B

	Control group (*N* = 30)	Treatment group (*N* = 30)	t*	*P* value
	Mean ± SD	Mean ± SD***		
Age	37.33 **±** 10.77	37.17 **±** 14.41	0.05	0.96
Follow up (months)	25.6 **±** 1.76	25.5 **±** 1.5	0.2	0.4
	**N**	**N**	**X** ^2**^	***P*** **value**
Sex (male/female)	23/7	23/7	0.00	1.00
Smoking (Yes/No)	19/11	20/10	0.07	0.79
Side (Rt./Lt.)	12/18	17/13	1.67	0.20
MOT (RTA/Fall from height/Simple twist)	9/17/4	13/15/2	1.51	0.45
Fracture type (closed /open)	25/5	20/10	2.22	0.14
AO classification (C2/C3)	2/28	3/27	0.22	0.64
Associated injuries (Yes/No)	8/22	12/18	3.46	0.06

*Student t test **Chi square test (FE: Fisher Exact) *** Standard deviation

MOT: Mode of trauma

**Table 2:** Treatment description in both groups

		Control group (*N* = 30)	Treatment group (*N* = 30)		
		Mean ± SD	Mean ± SD		
Time for conversion		11 **±** 3	**-**		
External fixation time		**-**	21 ± 5		
Ankle spanning/sparing		**-**	27/3		
Mini-open / closed reduction		-	13/17		
		***N***	***N***	**X** ^2**^	***P*** **value**
Fibular fracture fixation	Not fractured	9 (30%)	6 (20%)	30	< 0.001
	IM fixation	1 (3.3%)	17 (56.7%)		
	Plates	20 (66.7%)	4 (13.3%)		
	Fractured not fixed	0	3 (10%)		

**Chi square test

IM: intramedullary

Primary outcome (Table 3).

The control group had significant higher AOFAS score where the mean was 85 ± 9 (range: 60–98) while it was 77 ± 10 (range: 55–95) in treatment group with a *P* value of 0.006.

Secondary outcomes (Table 3).

Regarding ankle range of motion (ROM) at the final follow-up, there was no considerable difference in mean dorsiflexion between control group (seven degree, range: 0–20°) and treatment group (five degree, range: 0–15°) with *P*-value equals 0.1. Similarly, the mean plantarflexion in control group was 33° (range: 25–45°), compared to 30° (range: 20–40°) in treatment group with *P*-value equals 0.06. The mean duration for returning to work was seven months (range: 5–12) in control group compared to ten months (range: 6–18) in treatment group with *P* value of less than 0.001.

There was no significant difference in the quality of reduction assessed at final follow between the two groups (*P*-value 0.14).

Regarding union time, control group exhibited a mean of 17 weeks (range: 12–30), while treatment group showed a mean of 19 weeks (range: 12–26) with *P*-value of 0.02.

In control group, no cases of nonunion were reported. However, two cases (6.7%) experienced delayed union, and another two cases (6.7%) presented with malunion, showing lateral distal tibial angles (LDTA) of 81.5° and 80°, respectively. In contrast, treatment group had three cases (10%) of nonunion at the meta-diaphyseal junction. These were managed with bone edges refreshment, autologous bone grafting, and extended fixation using an Ilizarov frame. Additionally, four cases (13.3%) experienced delayed union, while four cases (13.3%) developed malunion—one with LDTA of 80°, two with anterior distal tibial angles (ADTA) of 75°, and one with a diaphyseal malalignment, which required corrective osteotomy and Ilizarov fixation. Statistical analysis revealed no significant differences between the two groups regarding nonunion, delayed union, and malunion, with *P*-values of 0.24, 0.39, and 0.39, respectively.

In control group, PTOA was observed in two cases (6.7%), while treatment group presented three cases (10%) with PTOA. (*P*-value = 0.64)

In control group, three cases of FRI were reported, representing 10%. The first case developed early FRI within two weeks and was managed with debridement with metalwork retaining. The other two cases developed FRI at one and four months postoperatively, respectively. Both required debridement and metalwork removal; however, only the earlier case needed additional fixation using an Ilizarov frame in conjunction with a local muscle flap. The latter case did not require further fixation. In treatment group, four cases (13.3%) of FRI were identified. One patient developed wound necrosis five days postoperatively, requiring a skin graft. Another patient presented with a wound infection and was successfully treated with debridement only. The remaining two patients developed osteomyelitis (OM) following frame removal and were managed with debridement and sequestrectomy, without the need for further fixation. Statistical analysis revealed no significant difference in FRI rates between the two groups, with a *P*-value of 0.69.

Regarding the need for secondary procedures, three patients (10%) in control group required additional surgical interventions due to FRI. In comparison, seven patients (23.3%) in treatment group required further procedures, four for FRI and three for nonunion. (*P*-value = 0.16).

**Table 3:** Difference in main outcomes in both groups

		Control group (*N* = 30)	Treatment group (*N* = 30)	t*	*P* value	95% CI
		Mean ± SD	Mean ± SD			
Ankle ROM	Dorsiflexion	7 ± 5.7	5 ± 4	1.29	0.1	MD + 2.00 [− 0.55, + 4.55]
	Plantarflexion	33 ± 4	30 ± 3	1.59	0.058	MD + 3.00 [+ 1.17, + 4.83]
Time to union (weeks)		17 **±** 3.6	19 **±** 3.5	2.53	0.02	MD − 3.00 [− 4.24, − 1.76]
Return to work (months)		7 **±** 1.5	10 **±** 3	4.03	< 0.001	MD − 2.00 [− 3.83, − 0.17]
AOFAS		85 **±** 9	77 **±** 10	3.3	0.006	MD + 8.00 [+ 3.08, + 12.92]
		***N***	***N***	**X** ^2**^	***P*** value	**95% CI**
Post Operative Burwell and Charnley score	Anatomical	19 (63.3%)	14 (46.7%)	4.18	0.14	RD + 0.167 [− 0.082, + 0.415]
	Fair	10 (33.3%)	10 (33.3%)			RD 0.0 [− 0.239 to + 0.239]
	Poor	1 (3.3%)	6 (20.0%)			RD − 0.167 [− 0.323 to − 0.010]
FRI		3 (10%)	4 (13.3%)	0.16	0.69	RD − 0.033 [− 0.196, + 0.129]
Bone union	Non-union	0 (0%)	3 (10%)	3.16	0.24	RD − 0.100 [− 0.207, + 0.007]
	Delayed union	2 (6.7%)	4 (13.3%)	0.74	0.39	RD − 0.067 [− 0.218, + 0.084]
	Malunion	2 (6.7%)	4 (13.3%)	0.74	0.39	RD − 0.067 [− 0.218, + 0.084]
PTOA		2 (6.7%)	3 (10%)	0.21	0.64	RD − 0.033 [− 0.173, + 0.106]
Secondary procedure		3 (10%)	7 (23.3%)	1.92	0.16	RD − 0.133 [− 0.319, + 0.052]

*Student t test **Chi square test

CI: confidence interval

MD: Mean difference

RD: Risk difference

ROM: range of motion

AOFAS: American orthopedic foot and ankle society

FRI: fracture related infection

PTOA: post-traumatic osteoarthritis

## Discussion

This prospective randomized controlled study provides a comparative analysis between two-stage ORIF and single-stage LIFEF in the management of AO/OTA type C pilon fractures. It is important to note that previous studies comparing these techniques were predominantly retrospective, with only two randomized controlled trials (RCTs) conducted by Wang et al. [7] and Elgawadi et al. [8]. However, these RCTs included both type B and C fractures in their analyses. Therefore, to the best of our knowledge, this study stands as the first RCT specifically comparing these two methods in the management of complex pilon fractures (type C).

In this study the follow-up was complete for all randomized patients with a minimum duration of 24 months, reducing the risk of attrition bias and allowing assessment of medium-term outcomes. Regarding the quality of reduction at the final follow-up, there was no significant difference between the two groups which could be attributed to the use of mini open approaches in LIFEF cases, where articular reduction using ligamentotaxis alone failed.

The control group had higher ankle ROM, better AOFAS score and earlier return to work compared to treatment group. This could be explained by the fact that the majority of treatment group (90%) had ankle spanning constructs, which can delay ankle ROM, in contrast to control group, who were encouraged to start ROM as early as possible. These results are similar to the study by Blauth et al. [9], which stated that ankle function can improve with early motion and partial weight-bearing, providing a better chance for nutrition of the articular cartilage and recovery.

There was no significant difference in the incidence of FRI between the two groups. This is similar to the results of most other studies [7, 10–14]. Regarding bone healing complications, the current study found no significant differences between the two groups in the incidence of delayed union, nonunion, or malunion. Although malunion rates were slightly higher in treatment group, this is often attributed to loss of reduction resulting from inadequate fixation. Notably, all malunion cases had ankle-spanning constructs, which have been associated with higher rates of malunion compared to ankle-sparing constructs, as reported by Papadokostakis et al. [15]. Furthermore, patients in control group demonstrated a significantly shorter healing time compared to those in the treatment group, likely due to superior fixation stability in this group.

In contrast to the findings of Wang et al. [7], who reported no significant difference in healing time between the two groups, this study demonstrated a significant difference, with longer union times observed in treatment group. This may be attributed to the higher degree of fracture comminution in treatment group, which likely led to inadequate fixation and prolonged healing. Additionally, Wang et al. reported a higher rate of superficial infection in Ex Fix group, as they included pin tract infections in that category.

Regarding Elgawadi et al. [8] RCT, they found no significant differences in functional outcomes at nine months based on AOFAS scores and observed a shorter time to union in the LIFEF group. The improved outcomes in their study may be due to the use of ankle-sparing devices, which facilitated earlier ankle ROM. However, in our study, fracture complexity necessitated ankle-spanning devices in most cases (90%) to ensure stability, thereby limiting early mobilization. Moreover, the shorter union time in Elgawadi et al.’s study may be explained by their consistent use of closed reduction, whereas mini-open approaches were required in 43.3% of cases in our study.

The limitations of this study include that the relatively small sample size, which limits precision and external validity. Second, blinding of clinical outcome assessors was not feasible due to the visible differences between the interventions, introducing potential detection bias; only data analysts were blinded.

## Conclusion

In this study, two-stage ORIF was associated with better functional outcomes, shorter time to union and earlier return to work, with comparable reduction quality and complication rates relative to single-stage LIFEF.

## Data Availability

No datasets were generated or analysed during the current study.
